# Paraneoplastic Motor Neuropathy in Nodular Sclerosis Hodgkin’s Lymphoma: Case Report

**DOI:** 10.1155/crh/9468544

**Published:** 2026-04-30

**Authors:** Jayesh Menon, Tyler Rehbein, Patrick Reagan, Carla Casulo

**Affiliations:** ^1^ University of Rochester School of Medicine, Rochester, New York, USA; ^2^ Department of Neurology, University of Rochester, Rochester, New York, USA, rochester.edu; ^3^ Division of Hematology and Oncology, James P. Wilmot Cancer Center, University of Rochester Medical Center, Rochester, New York, USA, rochester.edu

## Abstract

Paraneoplastic neurological syndromes (PNS) are an uncommon complication of malignancy, characterized by autoantibody generation against neuronal structures, causing a spectrum of neurological syndromes affecting the central, peripheral, and/or autonomic nervous systems. PNS in lymphoma is exceedingly rare and there is limited evidence on the features, incidence, and severity of these neurological sequelae secondary to malignancy. Here, we report on a case of isolated diffuse motor neuropathy secondary to underlying nodular‐sclerosis classical Hodgkin’s lymphoma (cHL) in a 71‐year‐old male. This patient initially presented to the hospital with 3 months of progressive generalized weakness, weight loss, gait instability, and a rash. A broad workup revealed evidence of diffuse lymphadenopathy, which was characterized as nodular‐sclerosis cHL on biopsy. Concurrent neurological workup demonstrated generalized axonal motor neuropathy on nerve conduction studies with negative serologies for a panel of paraneoplastic autoantibodies, but positive Asialo‐GM1 titers, a marker of autoimmune myopathy. The patient was initiated on doxorubicin, vinblastine, and dacarbazine (AVD) for their lymphoma and received two doses of IVIg for their motor neuropathy, which did not reoccur. The patient was transitioned to brentuximab + nivolumab for 4 cycles, followed by nivolumab + AVD for 6 cycles with a complete response and no evidence of disease. Asialo‐GM1‐associated isolated diffuse motor neuropathy in Hodgkin’s lymphoma has not been previously reported in the literature. In this case report, we review the 2021 updated diagnostic criteria for the diagnosis of paraneoplastic neurological syndromes in the context of this patient presentation to highlight an instance where these guidelines do not fully capture the wide spectrum of PNS.

## 1. Background

Paraneoplastic neurological syndromes (PNS) are a group of conditions wherein aberrant production of autoantibodies in the setting of malignancy mounts an immune response against neuronal structures. The spectrum of PNS presentations have been previously characterized into distinct phenotypes stratified based on the frequency in which the underlying autoantibodies occur in malignancy. Autoantibodies which have been associated with malignancy in greater than 70% of cases are termed high‐risk; whereas, intermediate‐risk autoantibodies have been associated with cancer in 30%–70% of cases, and low‐risk autoantibodies were associated with a malignancy in less than 30% of patients. Among the high‐risk autoantibodies, 7 distinct phenotypes of PNS presentation have been identified: Encephalomyelitis, limbic encephalitis, rapidly progressive cerebellar syndrome, opsoclonus‐myoclonus, sensory neuronopathy, gastrointestinal pseudo‐obstruction, and Lambert–Eaton myasthenic syndrome. Phenotypes associated with intermediate risk autoantibodies include autoimmune or brainstem encephalitis polyradiculopathy, myelopathy, stiff person syndrome, myasthenia gravis, or Morvan syndrome, which is characterized by hyperactivation of the peripheral, central, and/or autonomous nervous systems secondary to an autoantibody against voltage‐gated potassium channels. Low‐risk PNS phenotypes are less defined, as the associated autoantibodies can frequently occur outside the setting of malignancy [1, 2].

PNS are relatively rare, and as such, the true incidence of PNS is not well understood but is estimated to be between 0.32 and 8.9 per 100,000 person‐years. In one longitudinal population‐based epidemiological study in Northern Italy encompassing 89 patients, the most common malignancy associated with PNS was lung cancer (17% of patients), followed by breast cancer (16%) and lymphoma (12%) [2, 3]. A subsequent epidemiological study in France reported similar trends in the relative frequency of PNS among different types of malignancy, with an incidence rate of 0.32 per 100,000 person‐years among a cohort of 632 patients, with lung cancer being the malignancy most commonly associated with PNS (24% of patients), followed by gynecological malignancies (14%), breast cancer (12%), lymphoma (5%), and renal cell carcinoma (4%) [4].

PNS is a rare complication of lymphoma. Given the limited number of such cases, large epidemiological studies have not been performed to date, and the existing literature on PNS in lymphoma largely relies on case reports/series and meta‐analyses. One foundational study, which examined cases of PNS in a cohort of 53 lymphoma patients across 20 European centers between 2000 and 2008, demonstrated that cerebellar degeneration occurred in 21 patients and was the most frequently occurring PNS among the study group. Demyelinating neuropathies were less frequently observed, with 11 patients among the cohort presenting with this form of PNS. Interestingly, the onset of PNS among this cohort occurred more frequently in advanced stages of disease. Autonomic dysfunction, as evidenced by hypothermia, was reported in 17 patients with advanced‐stage lymphoma; whereas this clinical finding was not evident in any patients with early‐stage disease [5]. Reoccurrence of PNS during lymphoma relapse may be a common presentation based on findings from a recent meta‐analysis of 115 publications containing 85 patients, wherein 10 of the 13 patients in the cohort who relapsed re‐presented with their prior paraneoplastic syndrome [6]. Given the scarcity of cases and standardized diagnostic criteria for PNS in lymphoma, attributing the sequelae of a neurological syndrome to an underlying lymphoma relies largely on clinical judgment.

## 2. Case Presentation

T.M. is a 71‐year‐old male with hypertension, hyperlipidemia, T3aN0 prostatic adenocarcinoma in remission, and a history of tobacco use who presented to the emergency department after having two mechanical falls in the setting of a 3 month history of progressive generalized weakness, 20‐pound weight loss, and gait imbalance. During this time, he had several prior emergency department presentations for malaise and was found to have a worsening microcytic anemia, hyperbilirubinemia, and transaminitis during these encounters.

On initial presentation, the physical exam was notable for an ill‐appearing elderly male with scleral icterus, a V‐shaped rash around the neck, and dry, scaly patches on the elbows, scalp, and knees with purple plaques overlying the elbows and knees (Figure 1). Reduced muscle bulk in biceps and triceps and intrinsic hand muscle weakness were noted bilaterally, without spasticity or rigidity. Objective weakness was noted in several muscle groups (Table 1). Notably, the sensory and cranial nerve exam was unremarkable.

FIGURE 1Dermatological exam. Dermatological findings on presentation were most notable for (a) lichenification of bilateral elbows (left arm pictured), (b) ichthyosis of the scalp, (c) bruising and scaling of bilateral knees, and (d) mild violaceous rash overlying the dorsal aspect of both hands, concerning for Groton’s sign, a manifestation of dermatomyositis.

**Figure:**
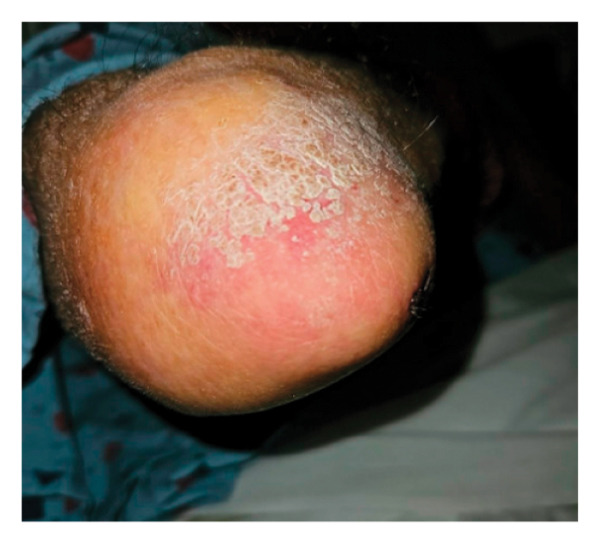
(a)

**Figure:**
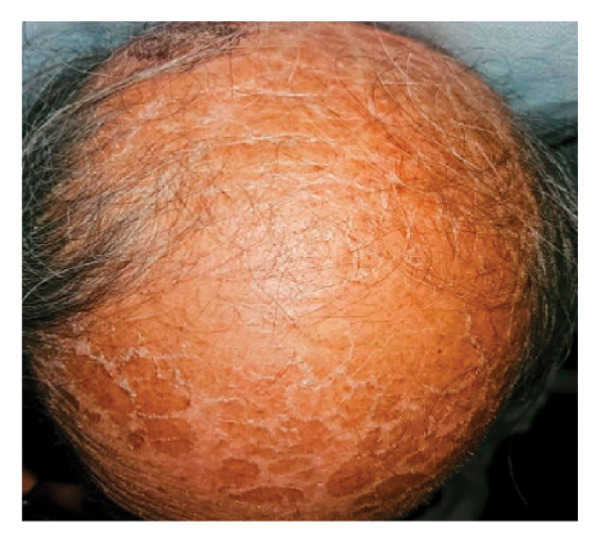
(b)

**Figure:**
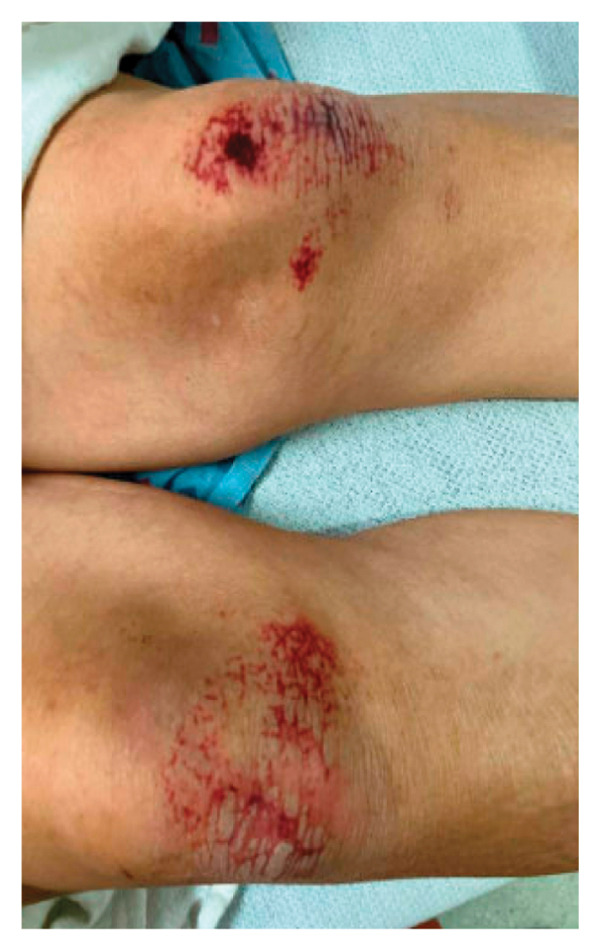
(c)

**Figure:**
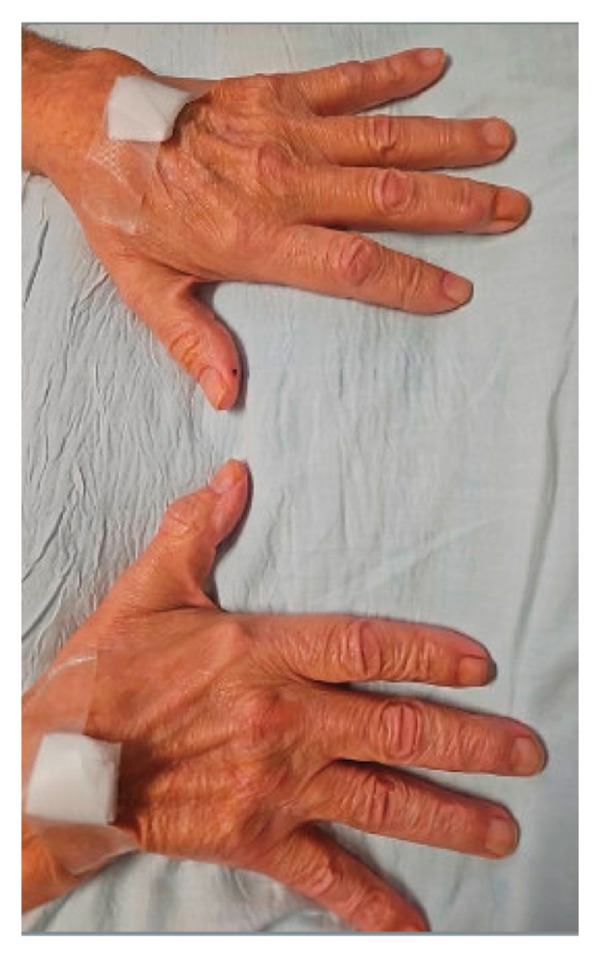
(d)

**TABLE 1:** Motor strength exam on presentation.

Muscle	Left	Right
Shoulder abduction	4	4
Elbow flexion	4	4
Elbow extension	4	4
Wrist flexion	5	5
Wrist extension	5	5
Thumb abductors	4−	4−
Finger abductors	4+	4+
Finger flexion	5	5
Hip flexion	4	4
Knee flexion	5	5
Knee extension	4	4
Ankle dorsiflexion	4	4
Ankle plantarflexion	5	5

*Note:* Strength graded on a scale out of 5 with “−” indicating slightly diminished strength and “+” indicating slightly increases strength.

Admission laboratories were notable for an ALT of 17 U/L (Ref: 0–50 U/L), AST of 217 U/L (Ref: 0–50 U/L), direct bilirubin of 1.9 mg/dL (Ref: 0.0–0.3 mg/dL), hemoglobin of 8.7 g/dL (Ref: 12–17 g/dL), platelets of 87 thousand/μL (150‐450 thousand/μL), and a lactate dehydrogenase of 658 U/L (Ref: 118–225 U/L). Creatine kinase (CK) was initially elevated at 1002 U/L (Ref: 39–308 U/L), but repeat CK preformed a few hours after presentation was within normal limits at 95 U/L. An elevated CK in the setting of a rash and subacute to chronic weakness was initially concerning for dermatomyositis secondary to possible malignancy or infectious and rheumatological etiologies; however, subsequent normalization of CK within hours of presentation was reassuring against an underlying myositis. Consultation was requested from the hematology/oncology, neurology, and rheumatology services for further workup.

To evaluate his generalized weakness, a myositis panel and skin biopsies were obtained, which were unremarkable for evidence of dermatomyositis; however, the skin biopsy demonstrated follicular spicules—a finding associated with multiple myeloma, human polyomavirus, and rarely, cutaneous lymphoma [7–9]. Further myeloma workup was initiated with serum protein electrophoresis with immunofixation, serum IgA, IgG, IgM, and kappa‐lambda free light chain quantification, all of which were unremarkable. Concurrent neurology workup was unrevealing for a structural brain or spinal lesion contributing to the patient’s weakness and gait abnormalities.

The CT neck, chest, and abdomen were performed to assess the possibility of a paraneoplastic process. This demonstrated diffuse lymphadenopathy, concerning for hematological malignancy (Figure 2). Bone marrow biopsy and lymph node biopsy were preformed, demonstrating classical Hodgkin’s lymphoma, nodular sclerosing subtype, with bone marrow involvement. Lymphoma staging imaging showed Stage IV disease.

FIGURE 2Diffuse lymphadenopathy noted on initial imaging. (a) Right and left hilar lymphadenopathy noted on initial CT chest, measuring 24 mm by 16 mm and 16 mm by 7 mm, respectively. (b) Right bulky cervical lymphadenopathy was noted within the cervical chain on a subsequent CT head and neck with a soft tissue protocol, with pictured nodes measuring 15 and 14.7 mm. (c) CT abdomen demonstrated portal adenopathy with the largest node measuring 19 mm by 27 mm.

**Figure:**
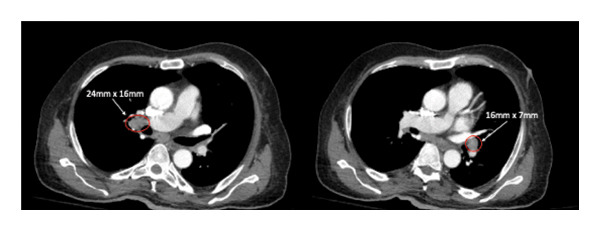
(a)

**Figure:**
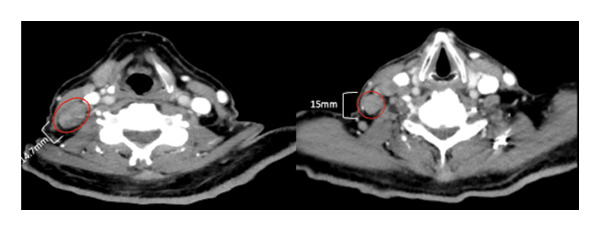
(b)

**Figure:**
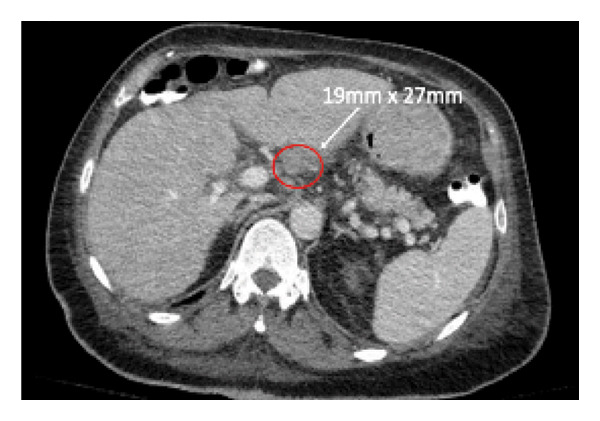
(c)

To investigate the relatedness of his weakness with the new diagnosis of Hodgkin lymphoma, nerve conduction studies were preformed, revealing a generalized axonal motor neuropathy. Needle EMG showed that in addition to expected chronic re‐innervation changes, some proximal muscles demonstrated features concerning for a myopathic process. Notably, compound muscle action potential (CMAP) amplitudes were diffusely decreased in upper and lower extremities, while sensory nerve action potential (SNAP) amplitudes and velocities were conserved. This needle EMG study was technically challenging and was repeated in the outpatient setting approximately 6 weeks later. On repeat testing, nerve conduction studies again demonstrated a generalized axonal motor neuropathy with similar needle EMG findings with evidence of new proximal upper extremity myopathic changes. Additionally, repetitive nerve condition stimulation testing was within normal limits during this examination. In both nerve studies, no areas of conduction block were noted.

An autoimmune myopathy panel was ordered, and serologies were negative for autoantibodies against HMG‐CoA, CN1a, MAG, voltage‐gated calcium channel, musk, acetylcholine receptor, LRP4A, and most surveyed ganglioside epitopes. Notably, the ganglioside antibody, the Asialo‐GM1 antibody, resulted in a positive at 62 IV (ref: < 51 IV – negative). Additionally, an autoimmune/paraneoplastic antibody panel was ordered but was unremarkable (Table 2). Given high clinical suspicion for a paraneoplastic motor neuropathy with a positive ganglioside autoantibody, the patient was placed on 2 g/kg of intravenous immunoglobulin (IVIg) over 5 days. The patient’s weakness began to rapidly improve after initiation of a loading dose of IVIg, and they deferred recommended maintenance IVIg infusions. The patient presented with a similar pattern of weakness approximately 2 months later and was initiated on a regimen of doxorubicin, vinblastine, and dacarbazine (AVD) for treatment of their Hodgkin’s lymphoma. During this hospitalization, they also received an additional dose of IVIg, which once again resulted in rapid resolution of symptoms, approximately 1 week after their first dose of AVD.

**TABLE 2:** Paraneoplastic antibody panel.

Antiantibody	Result
Amphiphysin	Negative
ANNA‐1	Negative
ANNA‐3	Negative
AGNA‐1	Negative
CASPR2‐IgG	Negative
CRMP‐5 IgG	Negative
GFAP	Negative
LGI1‐IgG	Negative
NIF	Negative
PCA Type I	Negative
PCA Type 2	Negative

T.M. subsequently continued treatment for their classical Hodgkin’s lymphoma following hospital discharge. Cycle 1 of AVD was complicated by an infusion reaction to dacarbazine and oliguria, and the patient was transitioned to brentuximab + nivolumab every 3 weeks for 4 cycles and achieved a complete response. After completion of brentuximab + nivolumab, the patient completed 6 cycles of nivolumab with AVD without evidence of disease. His Hodgkin’s lymphoma treatment was complicated by nivolumab‐associated hepatitis, which responded to a 1 month course of prednisone. At 1 year follow‐up from the initial diagnosis of this paraneoplastic motor neuropathy, the patient reports continued improvement in strength and gait without recurrence of their symptoms.

## 3. Discussion

Here, we report on a rare case of paraneoplastic axonal motor neuropathy in Hodgkin’s lymphoma. The incidence of weakness secondary to a paraneoplastic process in lymphoma is rare, with only a handful of paraneoplastic myositis cases described at the case report level [10, 11]. Interestingly, paraneoplastic dermatomyositis and polymyositis have been previously characterized in lymphoma [12]. Based on the needle EMG findings in our patient’s case, it is possible there may have been a myopathic component as well to his presentation. However, a rapidly normalizing CK level prior to any treatment is suggestive against myositis, and no muscle biopsy was performed due to his prompt improvement and deferral of further care. Another explanation for the mixed myopathic/neuropathic needle EMG findings could be “nascent units,” which can appear similar to myopathic changes on EMG but are neuropathic and associated with severe denervation and early incomplete reinnervation [13].

The diagnostic criteria for paraneoplastic syndromes, including paraneoplastic motor neuropathy, are based largely on the 2021 updated diagnostic criteria for PNS. These criteria classify PNS diagnoses based on clinical and laboratory findings into a numeric scoring system entitled PNS‐Care score. The PNS‐Care score is stratified into 4 categories of clinical suspicion—“definite”, “probable”, “possible”, or “non‐PNS”. In developing this scoring system, the presence of a well‐characterized PNS, including encephalomyelitis, limbic encephalitis, rapidly progressive cerebellar syndrome, opsoclonus‐myoclonus syndrome, sensory neuronopathy, gastrointestinal pseudo‐obstruction, or Lambert–Eaton myasthenic syndrome, was characterized as a “high‐risk” phenotype. “Intermediate risk” PNS phenotypes were defined as rapidly progressive (< 3 months) neurological syndromes that occur in the presence or absence of a malignancy. Additionally, the presence of autoantibodies against molecular patterns found within the nervous system is accounted for in the scoring system and is stratified into either “high‐risk” or “intermediate risk” (Table 3). These autoantibodies are well‐characterized in the literature, and those in the “high‐risk” category have been associated with malignancy in > 70% of cases (30%–70% in “intermediate‐risk”). The final component of the PNS‐Care score is the presence of an identified malignancy that is consistent with the observed PNS phenotype or autoantibody findings [2, 14].

**TABLE 3:** PNS‐Care score high and intermediate risk autoantibodies for any PNS.

Antiantibody	Risk
Hu (ANNA‐1)	High
CV2/CRMP5	High
SOX1	High
PCA2(MAP1B)	High
Amphiphysin	High
Ri (ANNA‐2)	High
Yo	High
Ma2/Ma	High
Tr (DNER)	High
KLHL11	High
AMPAR	Intermediate
GABAR	Intermediate
mGluR5	Intermediate
P/Q VGCC	Intermediate
NMDAR	Intermediate
CASPR2	Intermediate

Workup of this patient’s isolated axonal motor neuropathy was remarkable for a positive antiganglioside autoantibody, Asialo‐GM1, which is found within the myelin [15]. Autoantibodies against Asialo‐GM1 have been associated with several disease states, namely multifocal motor neuropathy (MMN), chronic inflammatory demyelinating polyradiculopathy (CIDP), and amyotrophic lateral sclerosis (ALS) [16, 17]. Interestingly, antibodies against Asialo‐GM1 have a demonstrated role within modulating the immune response. In a murine model, treatment of immune effector cells with receptors for Asialo‐GM1, namely NK cells and macrophages, resulted in ablation of NK cell populations and inhibition of the pro‐inflammatory response of macrophages [18]. Additionally, there is some evidence to suggest that anti‐Asialo‐GM1 antibodies may also deplete basophil populations, an immune cell with a key role in modulating the inflammatory response [19]. These findings suggest that there is an association with anti‐Asialo‐GM1 antibodies, immune dysregulation syndromes, like malignancy, and motor neuropathies. While the identified antiganglioside antibody, Asialo‐GM1, is not characterized as a high‐risk or intermediate risk autoantibody within the PNS‐Care model, the rapid progression of the patient’s weakness, in conjunction with needle EMG and nerve condition study findings concerning for motor neuropathy, likely places this presentation in the “intermediate‐risk” phenotype according to the PNS‐Care criteria. This characterization alongside the identification of a malignancy places this patient’s presentation into the “probable PNS” category within the PNS‐Care score stratification. The limiting factor preventing a “definite” diagnosis of PNS is the absence of a high‐ or intermediate‐risk antibody. While the association between anti‐Asialo‐GM1 antibodies and focal motor neuropathies has been well characterized, examination of this patient’s weakness revealed only axonal loss, with no conduction block or slowed velocities—findings that would be expected in the MMN associated with the presence of anti‐Asialo‐GM1 antibodies. This discordance between the expected findings of motor neuropathy secondary to anti‐Asialo‐GM1 autoantibodies, and this patient’s nerve studies complicates the diagnostic certainty, further precluding the classification of this presentation as “definite” within the PNS‐Care score stratification.

Here we report on an unusual isolated axonal motor neuropathy presenting with dermatological findings mimicking dermatomyositis in the setting of newly diagnosed classical Hodgkin’s lymphoma. This presentation is not well characterized within the current body of literature, with only a handful of case reports documenting a motor neuropathy or weakness associated with demyelinating disease secondary to Hodgkin’s disease [20–22]. Interestingly, to our knowledge, none of the cases presently described within the literature describe dermatological findings, not consistent with dermatomyositis, in association with underlying malignancy. In T.M.’s presentation, the presence of dermatological findings prompted a throughout workup of his generalized weakness. While resolution of his CK and negative skin biopsy indicated that a diagnosis of dermatomyositis was unlikely, these dermatological findings raised suspicion for some form of systemic illness, ultimately enabling a relatively quick diagnosis of Hodgkin’s disease and paraneoplastic axonal motor neuropathy, which responded excellently to IVIg monotherapy. While T.M. was undergoing outpatient workup for his persistent anemia, it was ultimately his progressive weakness that prompted hospitalization and further evaluation. Given the rare incidence of this paraneoplastic process and the difficulty in using existing diagnostics risk stratification tools to identify and classify these abnormal presentations, it is possible that cases of Hodgkin’s‐associated axonal motor neuropathy are initially missed or underdiagnosed. This case report serves to increase provider awareness of Hodgkin’s‐associated axonal motor neuropathy and consideration for atypical presentations of this process.

## Author Contributions

Dr. Carla Casulo had full access to all of the data in this study and takes complete responsibility for the integrity of the data and the accuracy of the data analysis.

## Funding

No funding was received for this manuscript.

## Disclosure

All authors have read and approved the final version of the manuscript.

## Consent

All the patients allowed personal data processing and informed consent was obtained from all individual participants included in the study.

## Conflicts of Interest

Patrick Reagan received institutional research funding from Pfizer. The other authors declare no conflicts of interest.

## Data Availability

The data that support the findings of this study are available from the corresponding author upon reasonable request.
